# Why use a mirror to assess visual pursuit in prolonged disorders of consciousness? Evidence from healthy control participants

**DOI:** 10.1186/s12883-017-0798-1

**Published:** 2017-01-24

**Authors:** Damian Cruse, Marco Fattizzo, Adrian M. Owen, Davinia Fernández-Espejo

**Affiliations:** 1 School of Psychology, University of Birmingham, Birmingham, B15 2TT England; 2 Department of Neurorehabilitation, University Hospital of Ghent, Ghent, 9000 Belgium; 3 The Brain and Mind Institute, University of Western Ontario, London, Ontario N6A 5B7 Canada

**Keywords:** Brain injuries, Consciousness disorders, Diagnosis, Smooth pursuit

## Abstract

**Background:**

Evidence of reliable smooth visual pursuit is crucial for both diagnosis and prognosis in prolonged disorders of consciousness (PDOC). However, a mirror is more likely than an object to elicit evidence of smooth pursuit. Our objective was to identify the physiological and/or cognitive mechanism underlying the mirror benefit.

**Methods:**

We recorded eye-movements while healthy participants simultaneously completed a visual pursuit task and a cognitively demanding two-back task. We manipulated the stimulus to be pursued (two levels: mirror, ball) and the simultaneous cognitive load (pursuit only, pursuit plus two-back task) within subjects.

**Results:**

Pursuit of the reflected-own-face in the mirror was associated with briefer fixations that occurred less uniformly across the horizontal plane relative to object pursuit. Secondary task performance did not differ between pursuit stimuli. The secondary task also did not affect eye movement measures, nor did it interact with pursuit stimulus.

**Conclusions:**

Reflected-own-face pursuit is no less cognitively demanding than object pursuit, but it naturally elicits smoother eye movements (i.e. briefer pauses to fixate). A mirror therefore provides greater sensitivity to detect smooth visual pursuit in PDOC because the naturally smoother eye movements may be identified more confidently by the assessor.

## Background

Visual pursuit of a moving object signifies the transition from unawareness to awareness in prolonged disorders of consciousness (PDOC) – i.e. from vegetative state to minimally conscious state [1]. Accurate differential diagnosis is crucial in PDOC because treatment decisions may be influenced by differences in likelihood of functional recovery that are associated with each diagnostic group [2, 3].

Two recent studies found that visual pursuit is significantly more likely to be observed in PDOC when the stimulus to be pursued is the patient’s own face reflected in a mirror, relative to when the stimulus is an object or person. [4, 5]. The cause of this effect is unclear, although it has been hypothesised that the patient’s own face is more likely to attract the gaze as it is a salient and auto-referential stimulus [4, 5]. This hypothesis, however, presumes that reflected-own-face pursuit indexes consciousness to the same extent as object pursuit. In other words, a patient who only pursues their reflected-own-face, and not an object, is considered to be conscious to the same clinical level as a patient who pursues both. Any difference in pursuit behaviour between these two patients is considered to stem from an attention deficit that can be overcome with a sufficiently salient stimulus [4, 5].

However, extremely highly learned stimuli, such as one’s own name or own face, can elicit complex responses in the absence of awareness. For example, one’s own name is subjected to complex neural processing even during non-REM sleep — a period in which an individual is demonstrably unaware [6]. Furthermore, the brain’s electrophysiological response differentiates one’s own-face from faces of others even when the individual is unaware of having seen a face at all [7]. An alternate interpretation, therefore, is that processing of high relevance stimuli, such as pursuit of one’s own reflected face, may be less reliant on overt consciousness than processing of less salient stimuli, such as pursuit of an object. In other words, is reflected-own-face pursuit less demanding than object pursuit, thus allowing patients with lower cognitive abilities, or even lower levels of consciousness, to exhibit reliable pursuit? This interpretation has important implications for diagnoses achieved through responses to high relevance stimuli, such as own-face and own-name [8–10]. A further alternative physiological interpretation for the difference in pursuit behaviour is that conscious patients are actively attempting to pursue both stimuli, but that the eye movements during reflected-own-face pursuit are naturally smoother and therefore easier for an assessor to identify confidently.

To test these hypotheses, we asked a group of healthy participants to separately pursue an object and their reflection in a mirror while completing a challenging secondary task designed to reduce the cognitive resources available for smooth pursuit. If reflected-own-face pursuit does not draw on conscious resources to the same extent as object pursuit, we would predict differential performance on the secondary task and on measures of smooth pursuit, quantified via a head-mounted eye-tracker.

## Methods

### Participants

We recruited 25 participants from the research pool of the University of Western Ontario (UWO). All participants reported normal vision and were compensated with course credit. We excluded data from 8 participants due to hardware malfunction (*n =* 3) or chance-level task performance (*n =* 5), leaving 17 participants (median age: 18-years, range 18–23). The Research Ethics Board of UWO approved this study.

### Equipment

**Fig. 1:**
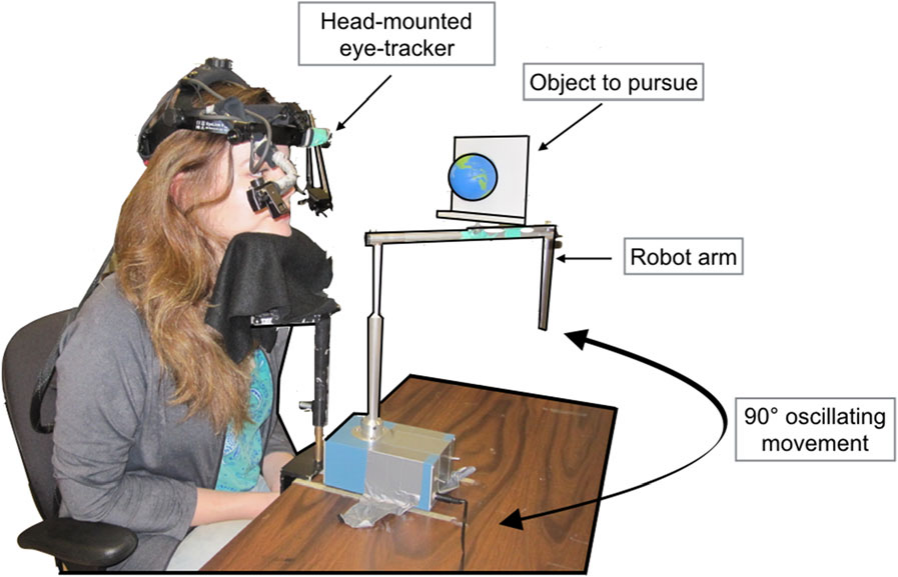
Experimental setup including eye-tracker, robot arm, and object to be pursued

A robot arm repeatedly moved objects through 90° on the horizontal plane ~30-cm in front of the participant’s eyes (45° either side of forward-facing; manufactured by Bonneville Scientific Inc., Salt Lake City, Utah, USA). One full oscillation lasted 10-s. An Eyelink II (SR Research Ltd, Mississauga, Ontario, Canada) tracked the movements of the left-eye (sample rate: 500-Hz; Fig. 1). The experimenter recalibrated the eye-tracker prior to each trial. The object stimulus (a ball) was mounted to the back of the mirror at the end of the robot arm, and the appropriate stimulus turned to face the participant on each trial. This ensured that the size of the moving portion of the arm was consistent across stimulus conditions. Prior to completion of mirror trials, the experimenter ensured that the mirror was placed in such a way that the participant could see the reflection of their face throughout the robot arm’s range of movement.

### Cognitive task

To increase cognitive load while participants pursued the stimuli, we developed a two-back category-matching task. Participants heard a series of spoken letters (3-s onset asynchrony) and indicated via button press whether or not each item was from the same category as that two items previously. Five letters that contain horizontal lines when written in their capital form comprised one category (E, F, H, L, T) and five that did not (V, W, X, Q, S) comprised the other. Such an orthographic comparison task was hypothesised to tax visual processing and interfere with pursuit demands. A pseudo-random series of 90 letters (22 matches) was created per trial.

### Design and procedure

Participants completed four trials from a two-by-two design — two stimuli (mirror, ball) x two levels of cognitive load (pursuit only, pursuit plus two-back) – in counterbalanced order. Prior to the experiment, participants completed a practice two-back task until they understood the procedure. Participants were instructed to smoothly pursue the moving stimuli with their eyes without moving their head. Each trial lasted 4.5-min followed by a brief rest.

### Analyses

To rule out potential task non-compliance, we excluded those participants with chance performance on either trial. Specifically, we shuffled the recorded button presses within trials and calculated the corresponding discrimination value (p[hit] minus p[false alarm] [11]), thus controlling for response biases. We performed this procedure 1000 times, creating a distribution under the null hypothesis that discrimination was not different from chance. Participants with discrimination within the lower 95% of the surrogate distribution (i.e. *p >* .05) were excluded from analyses (conducted with MATLAB).

Frequentist and equivalent Bayesian comparisons with default priors were conducted with JASP Version 0.7.1.12 [12, 13]. Specifically, to complement the t-tests, the Jeffrey-Zellner-Siow Bayes factor (JZS-BF_10_) tested the strength of the evidence for each observed effect size [14]. A JZS-BF_10_ ANOVA approach contrasted the strength of evidence for models reflecting the null, main effects, and interaction [15]. A JZS-BF_10_ between 1/3 and 3 is considered to be only weak/anecdotal evidence for an effect; 3–10: substantial evidence; 10–100: strong evidence; >100: very strong evidence [16]. The same category descriptions hold for the inverse.

## Results

### Cognitive task

Discrimination did not significantly differ between pursuit stimuli in a paired samples *t*-test (t(16) = .330, *p =* .746). A Bayesian equivalent indicated substantial evidence in favour of the null hypothesis (JZS-BF_10_ = .261). Mean reaction time did not significantly differ between pursuit stimuli in a paired samples *t*-test (t(16) = .866, *p =* .399). A Bayesian equivalent indicated marginally more evidence for the null hypothesis (JZS-BF_10_ = .346).

### Fixations

A two-way repeated measures ANOVA on numbers of fixations with factors of stimulus (ball, mirror) and cognitive load (pursuit only, pursuit plus two-back) revealed a marginal effect of load only (F(1,16) = 4.486, *p =* .050; stimulus: F(1,16) = .053, *p =* .821; interaction: F(1,16) = .004, *p =* .948). A Bayesian equivalent indicated that no model was preferred relative to the null model (all JZS-BF_10_ < .506).

**Fig. 2:**
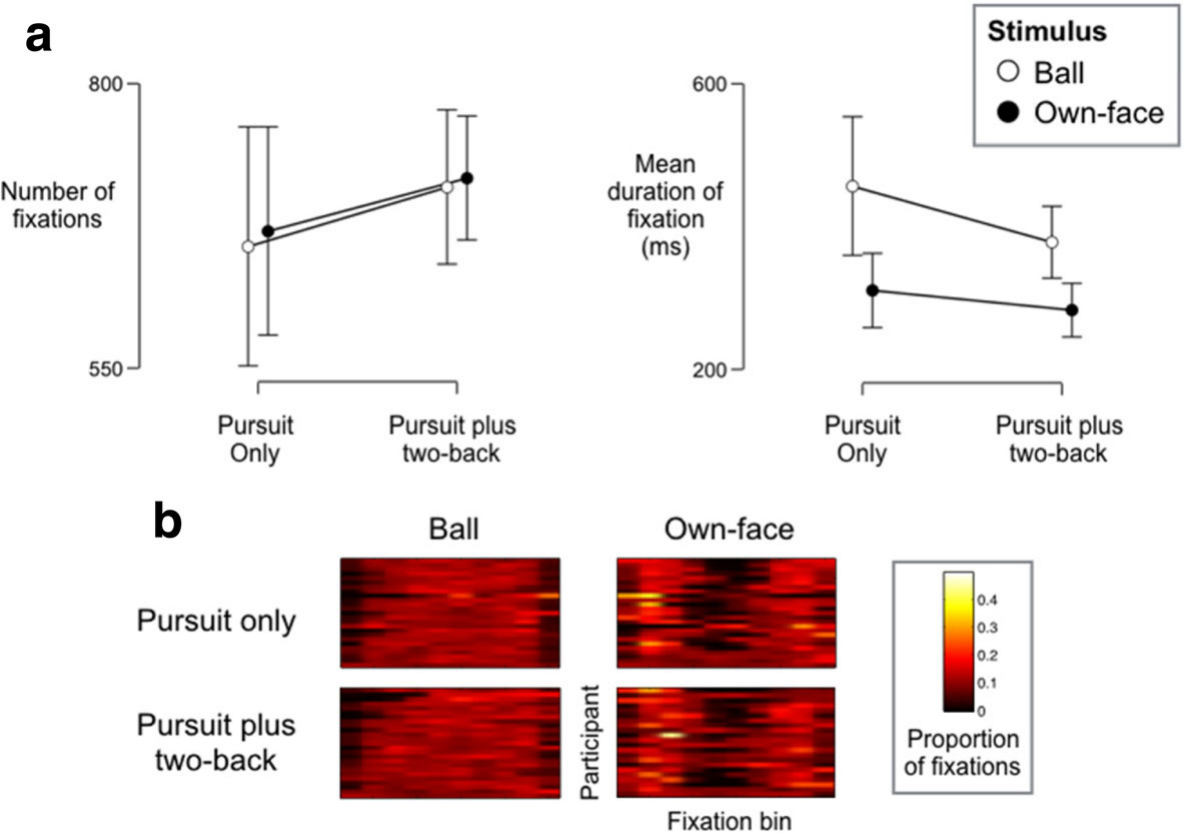
**a** Fixation behaviour across trials. Bars show 95% confidence intervals. **b** Single-subject distributions of fixations across trials. Each row of each panel shows data from one participant separated into ten bins across the horizontal plane of pursuit. Right-hand images highlight greater foci of fixations at the tails during reflected-own-face pursuit

A further ANOVA on average durations of fixation with the same factors revealed a significant effect of stimulus (F(1,16) = 15.763, *p =* .001), a marginal effect of load (F(1,16) = 3.294, *p =* .088), and no significant interaction (F(1,16) = .718, *p =* .409). A Bayesian equivalent indicated greater evidence for an effect of stimulus only, relative to all other models (JZS-BF_10_ = 121.430 relative to null). See Fig. 2a.

### Post-hoc analyses

During data quality checks, we noticed a qualitative difference in the spatial distribution of fixations (Fig. 2b). Specifically, fixations during object pursuit occurred relatively uniformly across the tracking space, while fixations during reflected-own-face pursuit occurred mostly when the mirror changed direction. To quantify this difference, we conducted the following post-hoc analysis. First, we removed outlier fixations (>2 standard deviations from mean horizontal or vertical coordinates). Next, we scaled the X-coordinates of fixations to be between 0 and 1, and tested this distribution against a uniform distribution (Chi-square goodness of fit, 10-bins). An ANOVA on the log-normalised Chi-square statistics with the same factors as above revealed a significant effect of stimulus only (F(1,16) = 14.299, *p =* .002; load: F(1,16) = .310, *p =* .585; interaction: F(1,16) < .001, *p =* .982). A Bayesian equivalent revealed substantial evidence for the model containing only the factor of stimulus relative to all other models (all JZS-BF10 > 3.735).

## Discussion

Our data indicate that pursuit of one’s own face reflected in a mirror is no less cognitively demanding than pursuit of an object, and therefore that both stimuli tap similar diagnostically important abilities in PDOC. Indeed, a Bayesian analysis indicates substantial evidence in favour of no differences in secondary task performance during reflected-own-face or object-pursuit.

Eye-tracking indicates that reflected-own-face pursuit is naturally smoother than object pursuit, and may therefore be less likely to be missed or confused with irrelevant staccato eye movements (e.g. nystagmus). Indeed, the data provide very strong evidence for significantly shorter mean eye fixations during reflected-own-face pursuit, which we interpret as a sign of smoother pursuit because the eyes do not take long pauses to fixate. Fixations also occur more uniformly across the space of object pursuit, consistent with less smooth pursuit.

While it is possible that the eye movements of healthy participants reported here do not generalise to PDOC, recent eye-tracking evidence suggests generalisability between patients and healthy controls [17]. We cannot rule out the possibility that a more demanding secondary task will have an effect on our measures of smooth pursuit, unlike the two-back task we employed here. However, even if this were the case, it is unlikely that the interaction between task performance and pursuit stimulus would be borne out, as indicated by the strong evidence against interaction models in our data (number of fixations: interaction JZS-BF_10_ = .040; duration of fixations: interaction JZS-BF_10_ = .404; relative to model with highest evidence). We therefore conclude that the cognitive demands of reflected-own-face pursuit and object pursuit are comparable.

These data provide further support for the use of a mirror in clinical assessments of awareness [4, 10]. It has been suggested that the greater sensitivity of the reflected-own-face stimulus stems from its higher salience and subsequent auto-referential processing – i.e. self-awareness [4, 5]. Indeed, there is evidence that autonomic responses to own-face photographs in the minimally conscious state are more similar to healthy individuals, while the autonomic response of patients in the vegetative state fails to differentiate between own-face and control stimuli [18]. Nevertheless, open questions remain regarding the subjective experience of patients who pursue their reflected own-face: does reflected-own-face pursuit index a simultaneous subjective experience of self-awareness on the part of the patient? Contemporary advances in brain imaging and physiological recordings may provide some insight into this question. Regardless, our data are consistent with the view that visual pursuit of reflected-own-face in PDOC is evidence of a similar level of consciousness and cognition to that identified by object pursuit, and therefore is a more appropriate method in clinical assessment.

## Conclusions

Our data provide substantial evidence that a mirror provides greater sensitivity to detect visual pursuit in patients with PDOC not because reflected-own-face pursuit is a less complex ability, but because the mirror naturally elicits smoother eye movements that can be identified more confidently by the assessor. These smoother and more reliable eye movements may be driven by the self-referential nature of the reflected-own-face stimulus, although further research is needed to appropriately characterise the relationship with subjective self-awareness.

## Abbreviations

ANOVA: Analysis of variance
JZS-BF: Jeffrey-Zellner-Siow Bayes factor
PDOC: Prolonged disorder of consciousness
REM: Rapid eye movement
